# Population scale analysis reveals gut microbiome diversity and functional shifts linked to obesity

**DOI:** 10.1016/j.isci.2026.117043

**Published:** 2026-09-17

**Authors:** Li-Fang Yeo, Joonatan Palmu, Aki S. Havulinna, Dattatray Mongad, Katariina Pärnänen, Veikko Salomaa, Leo Lahti, Rob Knight, Teemu Niiranen

**Affiliations:** 1 Division of Medicine, Turku University Hospital, Turku, Finland; 2 Department of Internal Medicine, University of Turku, Turku, Finland; 3 Department of Computing, University of Turku, Turku, Finland; 4 Research Program for Clinical and Molecular Metabolism, Faculty of Medicine, University of Helsinki, Helsinki, Finland; 5 Department of Microbiology, University of Helsinki, Helsinki, Finland; 6 Department of Public Health, Finnish Institute for Health and Welfare, Helsinki, Finland; 7 Department of Pediatrics, University of California, San Diego, La Jolla, CA, USA; 8 Center for Microbiome Innovation, University of California, San Diego, La Jolla, CA, USA; 9 Department of Computer Science and Engineering, University of California, San Diego, La Jolla, CA, USA; 10 Shu Chien-Gene Lay Department of Bioengineering, University of California, San Diego, La Jolla, CA, USA; 11 Halıcıoğlu Data Science Institute, University of California, San Diego, La Jolla, CA, USA; 12 Hong Kong University of Science and Technology Jockey Club Institute for Advanced Study, Hong Kong University of Science and Technology, Hong Kong SAR, China

**Keywords:** gut microbiome, obesity, cohort, cross-sectional

## Abstract

This study investigated the relationship between the gut microbiota and two obesity indicators, body mass index (BMI) and waist-hip ratio (WHR) in a well-phenotyped random population sample of 5846 participants. Associations were tested using multivariable-adjusted linear models and validated for BMI in an external cohort of 275 individuals. First, alpha diversity measured by Shannon was inversely associated with obesity but not observed species richness. Second, BMI was significantly associated with beta diversity (Bray Curtis) although there was no obvious clustering. Third, Bacteroidota:Firmicutes ratio was not associated with BMI or WHR. Finally, our study revealed that the gut microbiome of individuals with obesity was enriched with inflammation-associated species involved in lipid metabolism and pathways related to folate precursor, membrane lipid proteins and tRNA charging. Lean individuals were enriched in bacteria linked to cellulose breakdown.

## Highlights

• Alpha diversity (Shannon) was inversely associated with BMI
• Bacteroidota:Firmicutes ratio was not associated with BMI or WHR
• Individuals with obesity were enriched in inflammation-associated species
• Lean individuals were enriched in bacteria linked to cellulose breakdown

## Graphical abstract

**Figure:**
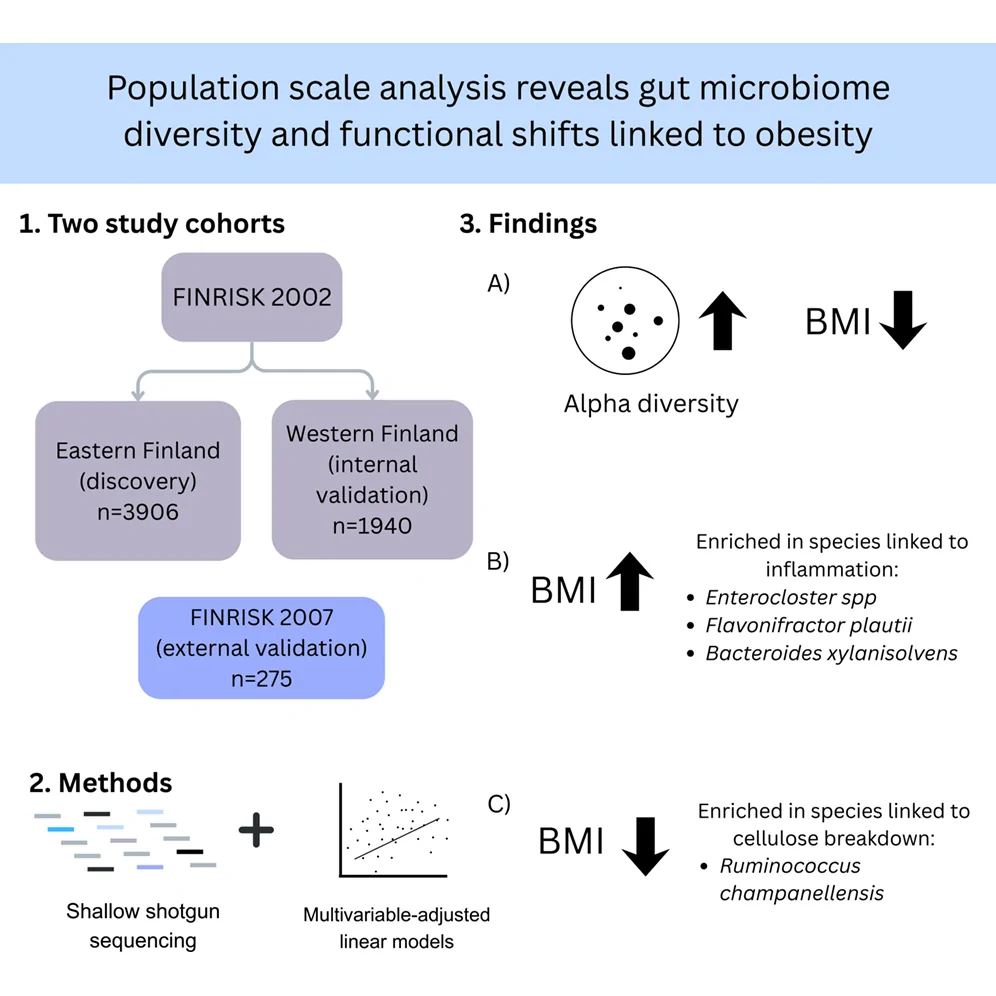

## Teaser

Health sciences; Obesity medicine

## Introduction

Obesity is a chronic condition with well-documented health risks, especially related to non-communicable diseases such as type 2 diabetes, cardiovascular disease, cancer, and mental health.^1^^,^^2^^,^^3^ In the early 2000s, mice studies demonstrated that the obesity phenotype could be transferred between mice via gut microbiota transplant and their associated metabolites.^4^^,^^5^ The link between gut microbiota and obesity has been intensively studied over the past decade across multiple research designs, involving epidemiological cohorts, animal studies, human intervention studies, and clinical trials.^4^^,^^6^^,^^7^^,^^8^^,^^9^^,^^10^

As gut microbiome studies gained traction globally, an influx of microbiome research also saw an increase in reports of conflicting results. To increase robustness, researchers very early on promoted consistent sample extraction methods, data generation, and pre-processing.^11^^,^^12^^,^^13^ Several attempts were subsequently made to reconcile the inconsistent results derived from gut microbiome and obesity studies.^14^^,^^15^^,^^16^ An overall consensus drawn from these studies was that (1) the alpha diversity of individuals with obesity as measured by Shannon was lower in obese individuals albeit with a relatively small effect size; (2) BMI was not associated with the overall community composition (beta diversity) and that there was no obvious clustering of obese vs. lean gut microbiomes; (3) Bacteroidetes:Firmicutes ratio was not associated with BMI despite previous claims.^14^^,^^15^^,^^16^^,^^17^^,^^18^

Furthermore, Sze & Schloss (2014) demonstrated that only one out of ten microbiome studies investigating links to BMI was sufficiently powered.^16^ A meta-analysis by Pinart et al.*,* (2021) and Díaz Perdigones et al.*,* (2025) reported that sample sizes of previous studies ranged from 20 to 1674.^17^^,^^18^ Consequently, previous studies have been limited by relatively low statistical power and lack of replication. Progress of translational microbiome studies was possibly also impeded by the use of early statistical models that were later considered to be inappropriate for the compositional nature of microbiome data, along with the lack of correction for confounders.^13^^,^^19^^,^^20^

In an effort to provide clarity over previous findings and new insights, we investigated the associations of gut microbiota and predicted metabolic pathways with obesity in an extensively phenotyped population cohort of 5846 participants. Multivariable-adjusted linear models were utilized and the findings were validated in an external cohort of 275 individuals.

## Results

The characteristics of the study participants of FINRISK 2002 are summarized in Table 1. The mean bacteria count of family, genus and species are listed in Data S1. The mean age of the participants was 48.5 ± 12.7 years and 53.7% were women.

**Table 1:** Characteristics table of study cohort

	Overall	Discovery cohort: Eastern Finland	Validation cohort: Western Finland
N	5846	3906	1940
Age, years (SD)	48.5 (12.7)	48.3 (12.7)	49.1 (12.9)
Women, n (%)	3141 (53.7%)	2095 (53.6%)	1046 (53.9%)
BMI, kg/m^2^ (SD)	26.8 (4.59)	27.0 (4.62)	26.5 (4.51)
Waist circumference, cm (SD)	89.3 (13.4)	89.4 (13.3)	89.1 (13.6)
Waist-hip ratio (SD)	0.90 (0.09)	0.90 (0.09)	0.89 (0.09)
Diabetes, n (%)	229 (3.9%)	163 (4.2%)	66 (3.4%)
Cardiovascular disease, n (%)	54 (0.9%)	41 (1.0%)	13 (0.7%)
Smoking, n (%)	1400 (23.9%)	921 (23.6%)	479 (24.7%)
Alcohol consumption, grams/week (SD)	83.1 (124)	72.8 (113)	104 (141)
Healthy food choices score (SD)^a^	197 (88.2)	193 (86.5)	203 (91.2)
Leisure-time exercise			
Light, n (%)	1198 (20.5%)	723 (18.5%)	475 (24.5%)
Moderate, n (%)	3274 (56.0%)	2253 (57.7%)	1021 (52.6%)
Vigorous, n (%)	1374 (23.5%)	930 (23.8%)	444 (22.9%)
Shannon diversity (SD)	4.12 (0.43)	4.11 (0.43)	4.13 (0.43)
Observed richness (SD)	1062.8 (215)	1064.6 (218)	1059.3 (210)
Total reads (SD)	1055378 (984488)	1061110 (991804)	1043837 (969742)

SD, standard deviation; BMI, body mass index.

a Higher healthy food choices score indicates more healthy food choices made per month.

### Alpha and beta diversity were overall associated with obesity

**Figure 1:**
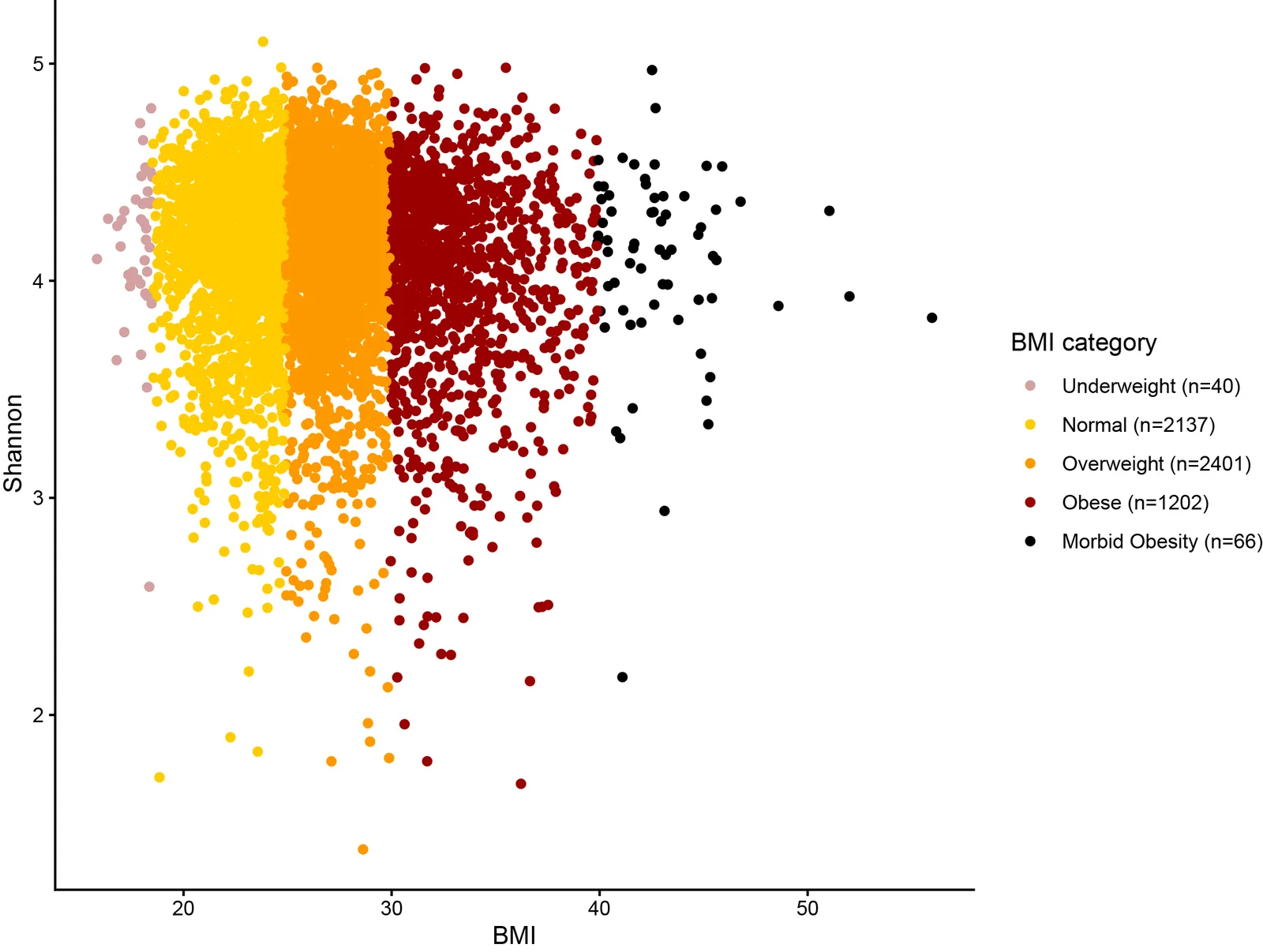
Alpha diversity by BMI category Alpha diversity (Shannon) was negatively associated with BMI using multivariable-adjusted linear model (−0.10 per one SD increase in BMI, 95% CI, 0.13 to 0.26, *p* = 3.7e−10). Linear model was adjusted for age, sex, diabetes, cardiovascular disease, healthy food choices, physical activity, smoking and alcohol consumption. BMI categories were used for visualization only. BMI categories were underweight < 18.5 kg/m^2^, normal 18.50–24.99 kg/m^2^, overweight 25–29.99 kg/m^2^, obese ≥ 30 kg/m^2^, morbid obesity ≥ 40 kg/m^2^.

**Figure 2:**
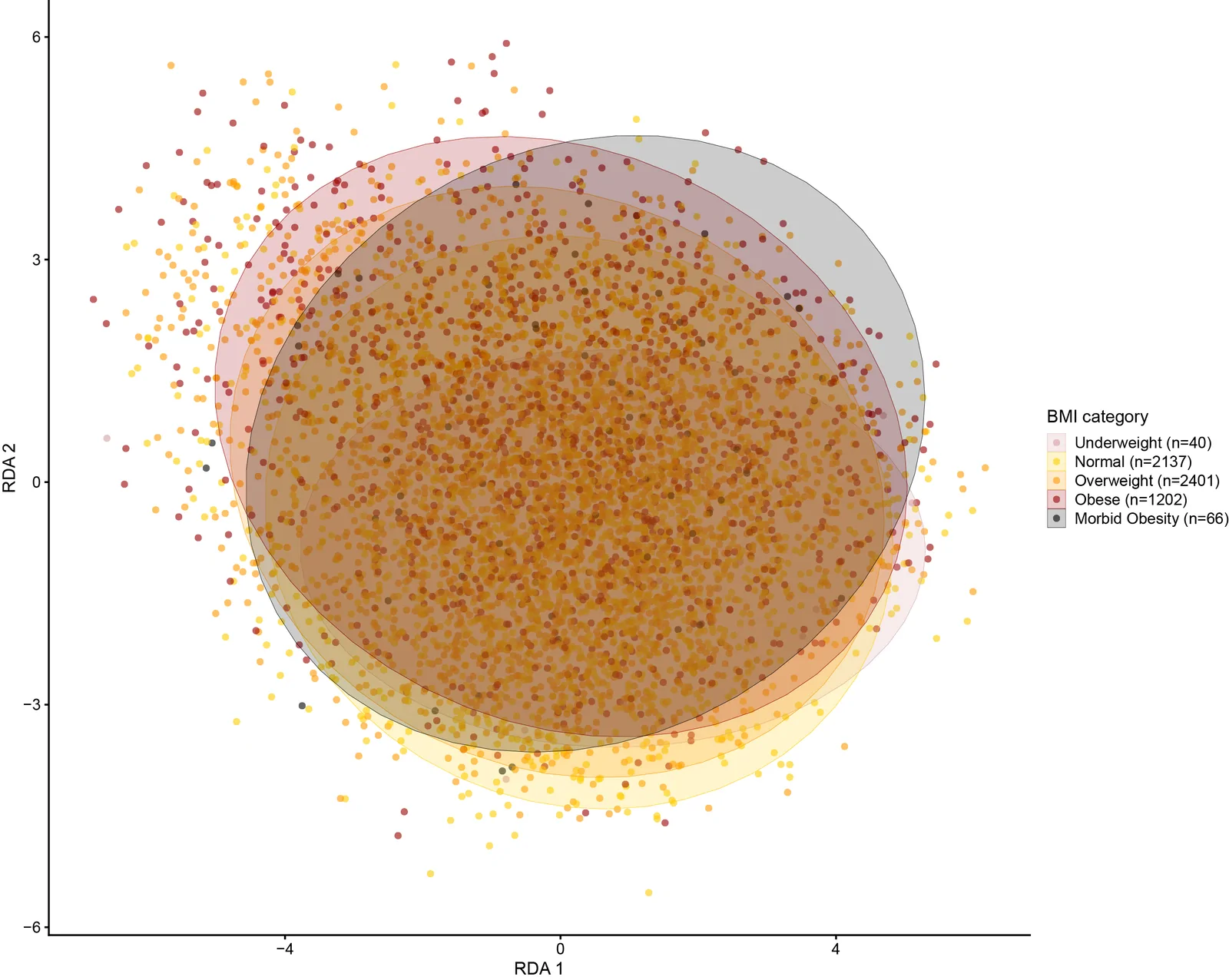
The relationship between microbiome composition and BMI Beta diversity was calculated with supervised ordination technique dbRDA (distance-based redundancy analysis), using the Bray-Curtis dissimilarity index at the species level. BMI was significantly associated and contributed to 0.3% variation in microbial composition. BMI categories were used for visualization only. BMI categories were underweight < 18.5 kg/m^2^, normal 18.50–24.99 kg/m^2^, overweight 25–29.99 kg/m^2^, obese ≥ 30 kg/m^2^, morbid obesity ≥ 40 kg/m^2^.

We found alpha diversity (Shannon index) to be negatively associated with BMI in the multivariable-adjusted models (−0.10 per one SD increase in BMI, 95% CI, 0.13 to 0.26, *p* = 3.7e−10, Figure 1). Observed richness was not significantly associated with BMI (0.01 per one SD increase in BMI, 95% CI, −0.01 to 0.04, *p* = 0.35). Shannon was inversely associated with WHR (*p* = 1.2e−06) but not observed microbial species index (*p* = 0.28, Tables S2 and S3). The multivariable-adjusted model explained 1.9% of the variation in microbial community structure (beta diversity, PERMANOVA, F-statistic = 11.45, *p* = 0.001, R^2^ = 0.019, Figure 2; Table S4). BMI contributed to 0.3% of this variation (*p* = 0.001, R^2^ = 0.003, Table S4). Homogeneity of covariates were tested and the dispersion for BMI was homogenous, supporting the validity of the results (F-statistic = 12.05, *p* = 0.89). The linear model with WHR explained 1.8% of microbial composition (beta diversity, PERMANOVA, F-statistic = 10.82, *p* = 0.001, R^2^ = 0.018, Table S5). However, homogeneity test for WHR was significant (F-statistic = 7.96, *p* = 0.001), suggesting that significance in beta diversity for WHR may be influenced by sample dispersion.

### Bacteroidota:Firmicutes ratio was not associated with obesity

Multivariable-adjusted linear models revealed that Bacteroidota:Firmicutes ratio were not associated with either BMI or WHR (BMI, 0.002 per 1-SD increase, *p* = 0.90; WHR, 0.005 per 1-SD increase, *p* = 0.58, Tables S6 and S7).

### Microbial abundances significantly different between lean and obese participants

Differential abundance analysis was performed by splitting the cohort internally into Eastern discovery cohort and Western validation cohort. Using multivariable-adjusted linear models, we found 164 and 149 bacterial species significantly associated with BMI and WHR, respectively, in the discovery cohort. Upon internal validation, 132 and 105 species remained significant for BMI and WHR, respectively (FDR <0.05, Figure S2). Many of the species were from the Oscillospiraceae, Lachnospiraceae, and Bacteroidaceae family (Data S2). As there were many common taxa associated with both BMI and WHR, subsequent analysis was performed only on BMI.

### Oscillospiraceae, Lachnospiraceae, and Bacteroidaceae remained significant in external validation

We used the FINRISK 2007 cohort to externally validate the internally validated species. FINRISK 2007 participants were younger, had lower BMI, had higher levels of physical activity, and consumed less alcohol than FINRISK 2002 participants (*p* < 0.05 for all, Table S8).

**Figure 3:**
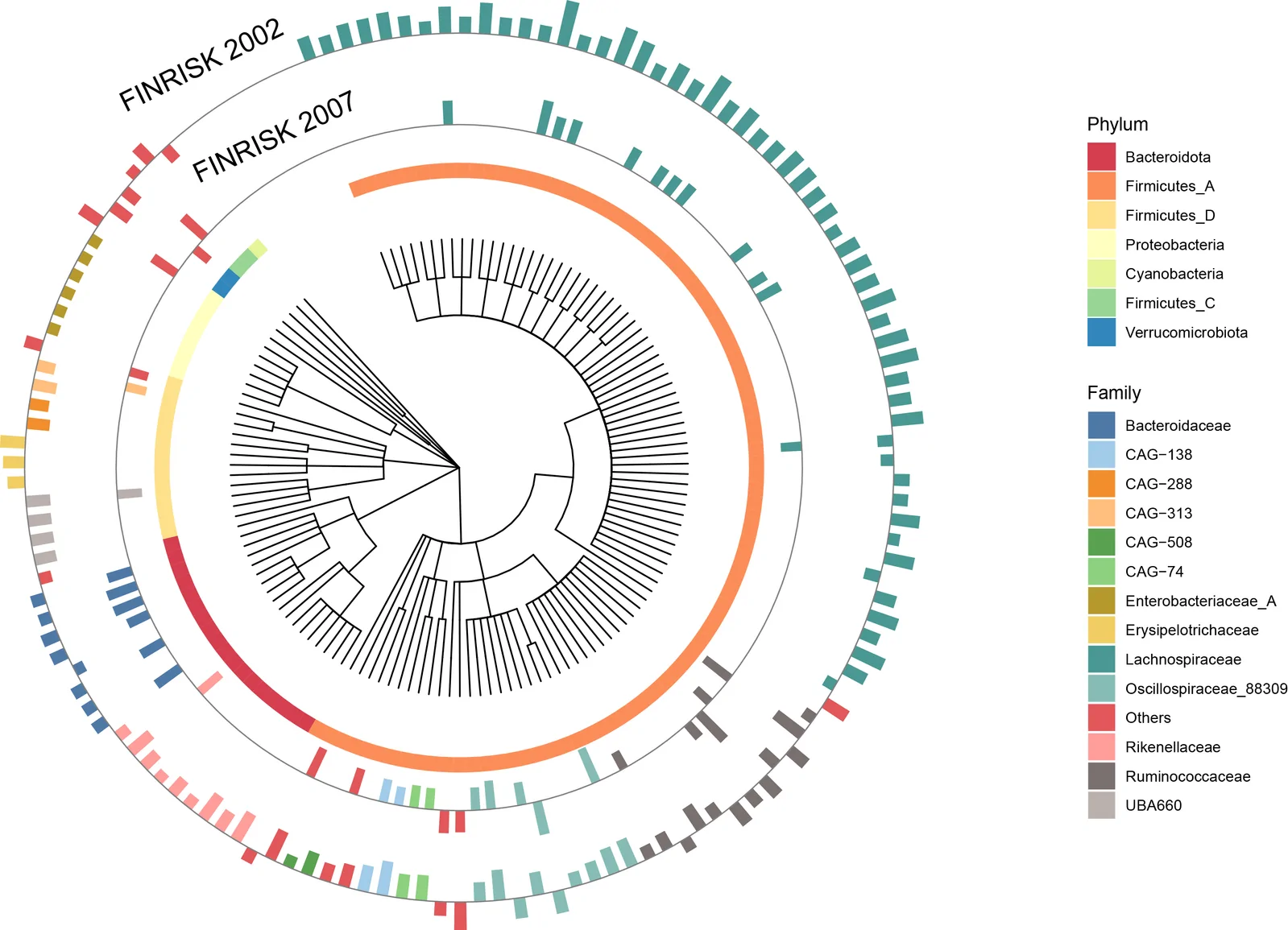
Taxonomic groups associated with BMI in FINRISK 2002 and validated in FINRISK 2007 The innermost circle depicts the phylum of the significant taxa. The bars on the two outer circles represent the linear model beta estimates per 1-SD changes in taxa abundance. The outermost circle depicts 132 species collapsed to the Family level that significantly associated with BMI using an internal validation (FDR <0.05). The inner circle depicts 43 species that remained significant in the external validation cohort, FINRISK 2007 (*p* < 0.05). Bacteria family that appears only once were labeled as “others”. Linear regression model was adjusted for age, sex, diabetes, cardiovascular disease, healthy food choices, physical activity, smoking, and alcohol consumption in FINRISK 2002 and age, sex, physical activity, smoking, and alcohol consumption in FINRISK 2007 external validation.

We tested 132 species that were significant in FINRISK 2002 and validated 43 species significantly associated with BMI (*p* < 0.05, Figure 3, Data S3). The direction of association was consistent with the FINRISK 2002 findings. The top ten most significant taxa are summarized in Table 2. The strongest associations were observed for the Oscillospiraceae family where *Neobittarella massiliensis* was positively associated with BMI (0.24 per 1-SD change in BMI, *p* < 0.001), and *Intestinimonas massiliensis* was negatively associated with BMI (−0.24 per 1-SD change in BMI, *p* < 0.001). *Enterocloster* sp000431375, *Flavonifractor plautii*, *Paraprevotella* sp003477995, *Phocaeicola* A 858004 *vulgatus*, *Bacteroides H xylanisolvens*, and *Dialister succinatiphilus* were positively associated with BMI, while *Ruminococcus F champanellensis* and *SFGY01 sp004556455* were negatively associated with BMI (*p* ≤ 0.001, Table 2).

**Table 2:** Top 10 BMI-associated species that were externally validated in FINRISK 2007

Family	Species	β	95% CI	P	FDR
Oscillospiraceae_88309	*Neobittarella massiliensis*	0.24	0.13–0.36	<0.001	0.004
Oscillospiraceae_88309	*Intestinimonas massiliensis*	−0.24	−0.36–−0.12	<0.001	0.004
Lachnospiraceae	*Enterocloster* sp000431375	0.23	0.11–0.34	<0.001	0.006
Oscillospiraceae_88309	*Flavonifractor plautii*	0.22	0.10–0.34	<0.001	0.009
Oscillospiraceae_88309	*Ruminococcus F champanellensis*	−0.21	−0.33–−0.10	<0.001	0.01
Prevotellaceae	*Paraprevotella* sp003477995	0.21	0.09–0.32	0.001	0.01
DTU023	*SFGY01* sp004556455	−0.20	−0.32–−0.09	0.001	0.01
Bacteroidaceae	*Phocaeicola* A 858004 *vulgatus*	0.20	0.09–0.32	0.001	0.01
Bacteroidaceae	*Bacteroides H xylanisolvens*	0.19	0.08–0.31	0.001	0.01
Veillonellaceae	*Dialister succinatiphilus*	0.19	0.08–0.31	0.001	0.01

β, beta estimate, CI, confidence interval; FDR, false discovery rate.

Linear regression model was adjusted for age, sex, physical activity, smoking and alcohol consumption. β estimates are reported as per 1-SD change in BMI and microbial abundances. FDR correction was not applied to the external cohort but as a sensitivity analysis the top ten species remained significant even after correcting for multiple testing (FDR ≤0.01).

### Obese microbiomes shifted toward energy harvesting and membrane biogenesis profiles

After dichotomizing the pathway variables, BMI was significantly associated with 24 pathways in the discovery cohort (FDR <0.05). Upon internal validation, only two pathways remained significant, namely tRNA charging (−0.39/1-SD, 95% CI, −0.58 to −0.20, FDR = 0.002) and L-histidine degradation (−0.35/1-SD, 95% CI, −0.54 to −0.16, FDR = 0.004). When inverse-rank normalized pathway data were used, a total of 124 predicted pathways were significantly associated with BMI. After internal validation, 61 predicted pathways remained significant (FDR <0.05, Data S4).

**Figure 4:**
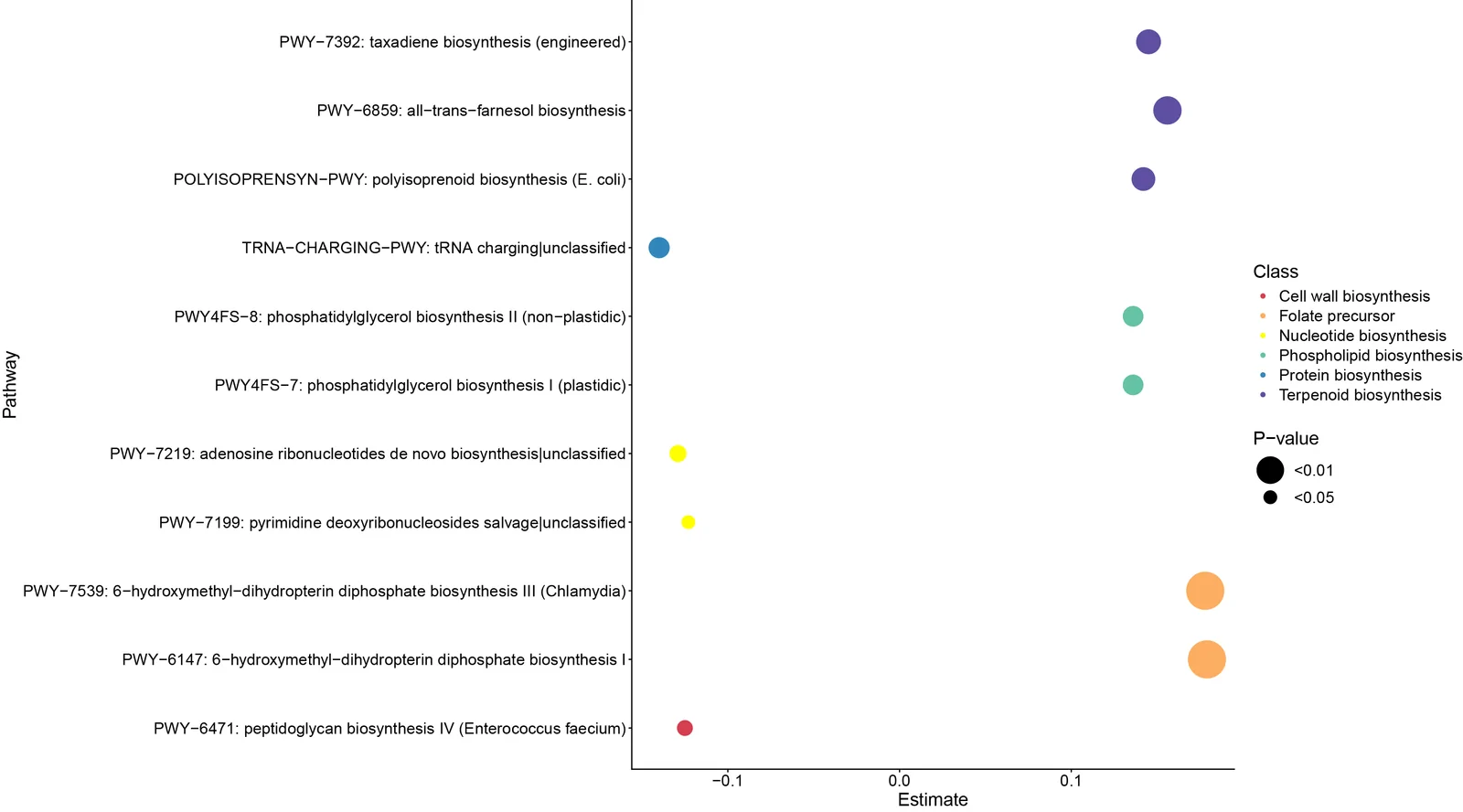
Predicted pathways significantly associated with BMI after external validation in FINRISK 2007 We externally validated 11 out of the 61 predicted pathways in FINRISK 2007 (*p* < 0.05). The strongest associations were related to folate precursor which synthesizes Vitamin B3. The other pathways were related to membrane lipid biosynthesis such as terpenoid and phospholipid, and also cell wall synthesis. Linear regression model was adjusted for age, sex, physical activity, smoking, and alcohol consumption.

Similar to the taxa abundance analysis, we used the FINRISK 2007 cohort to externally validate the 61 predicted pathways that were significantly associated with BMI in FINRISK 2002. We validated 11 out of the 61 pathways (*p* < 0.05, Figure 4). Folate precursor which synthesizes vitamin B3 was the pathway most strongly associated with BMI (*p* = 0.003). Pathways related to membrane lipids, such as terpenoid and phospholipid biosynthesis, were positively associated with BMI, while tRNA charging, nucleotide biosynthesis, and peptidoglycan biosynthesis were negatively associated with BMI (*p* < 0.05, Figure 4, Data S5).

### Approximately 89% of species-associated predicted pathways belong to ten species

As part of a sensitivity analysis, bacterial species significantly associated with each covariate (age, sex, diabetes, cardiovascular disease, healthy food choices, physical activity, smoking, and alcohol consumption), independent of BMI, was listed in a supplementary file for future investigation and improved interpretability (Data S6).

By assessing species-associated pathways, we found that 324 (dichotomized variables, pathway present/absent, and qualitative analysis) and 347 (inverse rank normalized, quantitative analysis) pathways were significantly associated with BMI after internal validation (FDR <0.05). Subsequent analysis was focused on 314 overlapping pathways that were significant in both qualitative and quantitative analyses. Most of the significant pathways could be attributed to a handful of species. The top ten species, namely *Eubacterium rectale*, *Bacteroides vulgatus*, *Ruminococcus torques*, *Butyrivibrio crossotus*, *Alistipes finegoldii*, *and Fusicatenibacter saccharivorans*, *Lachnospira pectinoschiza*, *Roseburia faecis*, *Roseburia inulinivorans*, and *Anaerostipes hadrus* account for 280 out of 314 (89%) pathways (Table S8).

Seven of the top ten species contributing to significant pathways overlapped with findings from the taxa differential abundance analysis. The direction of the linear models (enriched or depleted) was consistent for both taxa differential abundance and species-associated pathway analysis.

## Discussion

We utilized a large cohort to investigate the association between gut microbiome with two obesity indicators, BMI and WHR. The findings were further validated in an external cohort. Our study revealed that lean people had higher microbial diversity measured with Shannon index, which reflects both species richness and evenness. We did not observe a significant association of species richness with BMI and WHR, confirming several previous reports.^14^^,^^15^ A meta-analysis in 2014 assessed the association between gut microbiome alpha diversity and obesity and reported no consistent trend.^14^ Another systematic review in 2021 reported that nine out of 22 studies reported a significantly higher Shannon index among lean participants, which supports our findings.^17^ However, a subsequent meta-analysis of seven studies reported no significant differences.^17^ In a systematic review from 2025, six out of 16 studies reported significantly higher Shannon index among lean participants.^18^ Our findings are mainly in line with those presented by these previous studies, that is lean people tend to have higher microbial diversity as measured by Shannon index, but not species richness. According to calculations by Sze & Schloss (2016), our study with a sample size of 5846 individuals should be sufficiently powered to detect between-group differences of <1% in Shannon diversity.^16^ We also have a large sample size to confirm previous findings that obesity is not linearly associated with Bacteroidota:Firmicutes ratio.^14^^,^^21^

The two species with the strongest positive associations with BMI were *Intestinimonas massiliensis* and *Neobittarella massiliensis*. However, little is known of these species. We also observed that *Enterocloster* sp*000431375* was enriched in obese participants. In a clinical trial where 179 patients were given either placebo or a probiotic strain to treat chronic diarrhea, *Enterocloster* sp*000431375* was reported to be higher in patients with higher stress scores and became depleted after probiotic treatment.^22^ *Enterocloster* sp*000431375* was also reportedly increased in 88 children with allergic rhinitis induced by house dust mites compared to 50 healthy controls.^23^ Given that obesity is a state of low-grade inflammation, and that past studies seem to suggest that *Enterocloster* has been positively linked with inflammation, further studies could shed light the relation between this *Enterocloster* species and inflammation-associated diseases. We also found *Flavonifractor plautii* enrichment in obese participants. Previous studies have reported *F. plautii* to be involved in converting catechins, thereby reducing inflammation and protecting cells from oxidative stress.^24^ An *in vivo* and *in vitro* study reported a possible mechanism where *F. plautii* attenuated inflammation in adipose tissue by inhibiting TNF-α expression.^25^ However, our study revealed a seemingly opposite trend. We postulate that perhaps higher abundance of *F. plautii* in obesity may act as a countermeasure to the inflamed-state in obesity.

Our study also reported *Phocaeicola* A 858004 *vulgatus*, *Bacteroides H xylanisolvens*, and *Dialister succinatiphilus* in higher abundance among participants with obesity. *P. vulgatus* breaks down dietary fibers and produces the metabolite 3-hydroxyphenylacetic acid which is involved in lipid metabolism.^26^ Mice studies suggested *P. vulgatus* as a potential probiotic intervention for lipid metabolic disorder including diet-induced metabolic dysfunction-associated steatotic liver disease.^26^^,^^27^ However, when *Diaslister succinatiphilus* was co-colonized with *Phocaeicola* (*Bacteroides) vulgatus*, a succinate-producing bacterium, *D*. *succinatiphilus* reduced *P. vulgatus*-induced enhancement of colonic inflammation in oxazolon-induced colitis mice.^28^ It is thus possible that these species are affecting BMI together, instead of individually. Previous studies have also suggested that *Bacteroides xylanisolvens* has immunomodulatory capabilities and it appears to be involved in neuromodulation via the gut-brain axis. *B. xylanisolvens* has also demonstrated beneficial effects on the human host, such as tryptophan and folate production, and has been approved by the EU to be added to food.^29^^,^^30^ Lastly, our study revealed *Ruminococcus F champanellensis* and *SFGY01* sp004556455 as the only two species enriched in lean participants. *R. champanellensis* is a cellulose-degrading bacteria in the gut, commonly associated with high fiber diets and better cardiovascular health outcomes.^31^ On the other hand, not much is known about *SFGY01 spp.* The findings from our study, together with literature, seem to suggest enrichment in immunomodulatory and inflammation-associated bacteria that have been linked to lipid metabolism.

We further noted that *Akkermansia*, albeit being present in our dataset, was not significantly associated with either BMI or WHR. An early study with 10,534 participants from the American Gut Project reported otherwise.^32^ In a clinical trial, 32 overweight/obese volunteers administered *Akkermansia muciniphila* supplements exhibited overall improved metabolic parameters.^33^ A recent *in vitro* and *in vivo* study demonstrated potential pathways where *A. muciniphila* modulate host appetite regulation and lipid metabolism.^34^

Our pathway analyses revealed that the gut microbiome in individuals with higher BMI was enriched for folate precursor and membrane lipid biosynthesis. These findings are in line with our taxonomic level analysis where *B. xylanisolvens* was enriched in obese participants and simultaneously associated with folate precursor production. We also found that membrane lipid biosynthesis pathways were enriched in participants with obesity. We postulate that we are observing lipid bilayer stress during the pathogenesis of obesity-linked inflammation which results in modulation of membrane-lipid proteins.^35^ Furthermore, the tRNA charging pathway, which is a pathway essential for enabling amino acids to attach to transporter RNA molecules, was enriched in leaner individuals. The tRNA charging pathway encompasses bidirectional associations with fat metabolism and energy-related pathways in obesity and type 2 diabetes.^36^^,^^37^ Together with taxonomic and predicted pathway analysis, our study shows that the gut microbiome of individuals with obesity reflect enrichment in inflammation-associated species involved in lipid metabolism and pathways related to folate precursor, membrane lipid proteins and tRNA charging.

Next, a sensitivity analysis was conducted to investigate the concordance between taxonomic and functional associations. By limiting the analysis to predicted pathways associated with bacteria species, we observed 314 pathways significantly associated with BMI. Most of the significant pathways were enriched in sugar, amino acid, and nucleotide-related biosynthesis pathways. However, we acknowledge the limitations of this analysis and urge caution when interpreting these results. Firstly, the data were shallow sequenced which makes functional annotation challenging. As observed in our current data, the pathway counts were highly sparse. We mitigated this by applying both qualitative (absent/present) and quantitative (inverse-rank normalization) transformation on the data and reporting the results from both methods. We also observed that 89% of the significant pathways were assigned to ten species. We noted a high concordance between the taxonomic and pathway analyses as seven out of the ten pathway-associated species were also found significant in the taxonomic analysis. It is possible that the top significant pathways observed here are driven by the taxa themselves being strongly associated with BMI or, that the taxa are in higher abundance. The optimal method for mitigating this inherent limitation would be to perform targeted gut metabolomics in future studies. Henceforth, predicted pathway analysis, as reported in the main results, was conducted at a more general level, where species-associated pathways and non-bacteria pathways were removed.

### Limitations of the study

One of the strengths of this study was the large cohort size and the use of an internal and external validation cohort that was able to decrease false positives. We observed that after external validation, only the strongest predictors remained significant and the findings were highly coherent between taxa and predicted functions. Furthermore, the participants were drawn from the general population, thus reducing selection bias. Interestingly, the top ten strongest predictor species in the linear models were all anaerobes, which provide some confidence that there was minimal flora bloom despite the long storage before sequencing. However, we acknowledge some limitations in this study. Firstly, the FINRISK studies were designed to be representative of the Finnish general population, inadvertently limiting global generalizability. Nonetheless, past studies have showed that the overall microbiome composition of FINRISK participants is fairly similar to gut microbiomes of other cohorts from Western countries.^38^^,^^39^^,^^40^ Secondly, certain pathways or taxa may be underrepresented in shallow sequencing data. For example, *in vivo* and *in vitro* studies have demonstrated *Christensenella* spp to modulate host metabolism in association with obesity.^41^ However, we were unable to assess the associations between BMI and *Christensenella* spp. due to its low prevalence in our dataset. Thirdly, our linear models did not correct for metformin use, which has known effects on the gut microbiome.^42^ Instead, we adjusted for diabetes where a large proportion of diabetic individuals were metformin users. We decided not to include metformin use as a covariate to avoid collinearity. Finally, stool sampling was only performed at a single time point in year 2002. Repeated stool sampling and BMI measurements of the participants are currently underway and may provide new prospective and longitudinal insights in the near future.

## Resource availability

### Lead contact

Requests for further information and resources should be directed to and will be fulfilled by the lead contact, Li-Fang Yeo (lifyeo@utu.fi).

### Materials availability

This study did not generate new unique reagents.

### Data and code availability

• The FINRISK data for the present study are available with a written application to the THL Biobank as instructed on the website of the Biobank (https://thl.fi/en/research-and-development/thl-biobank/for-researchers).
• A separate permission is needed from FINDATA (https://www.findata.fi/en/) for use of the electronic health record data.
• Metagenomic data are available through the European Genome-Phenome Archive (EGAD00001007035).
• The source code and full list of R packages used for the analyses and figures have been deposited at GitHub and are available at https://github.com/finrisk2002/article-2026-gutmicrobiome-BMI-cross-sectional.
• Any additional information required to reanalyze the data reported in this paper is available from the lead contact upon request.

## Acknowledgments

We thank all participants of the FINRISK 2002 survey for their contributions to this work, and Tara Schwartz for assistance with laboratory work, and Kari Koponen for assistance with the Healthy Food Choices index. We also acknowledge the CSC-IT Center for Science, Finland, for computational resources. L.-F.Y. has received a research grant co-funded by the European Union's Horizon Europe Framework programme for research and innovation 2021-2027 under the Marie Skłodowska-Curie grant agreement No 101126611. J.P. has received research grants from the 10.13039/501100008484Paavo Nurmi Foundation and the 10.13039/100008723Finnish Medical Foundation. D.M. has received a research grant co-funded by the European Union's Horizon Europe Framework programme for research and innovation 2021-2027 under the Marie Skłodowska-Curie grant agreement no 101126611. K.P. has received grants (348439 and 368511) from the 10.13039/501100002341Research Council of Finland. V.S. has received a research grant from the 10.13039/501100004037Juho Vainio Foundation. T.N. received funding for this work from the Finnish Research Council, 10.13039/501100006306Sigrid Jusélius Foundation, 10.13039/501100005633Finnish Foundation for Cardiovascular Research, and The Wellbeing Services County of Southwest Finland.

## Author contributions

Conceptualization, V.S., L.L., R.K., and T.N.; data curation, J.P., A.S.H., D.M., K.P., V.S., L.L., R.K., and T.N.; formal analysis, investigation, visualization, writing, L.-F.Y., J.P., and T.N.; writing – review and editing; all funding acquisition, L.L., R.K., and T.N.

## Declaration of interests

R.K. is a scientific advisory board member, and consultant for BiomeSense, Inc., has equity and receives income. He is a scientific advisory board member and has equity in GenCirq. He has equity in and acts as a consultant for Cybele. The terms of these arrangements have been reviewed and approved by the University of California, San Diego in accordance with its conflict-of-interest policies. T.N. has received consulting fees from AstraZeneca.

## STAR★Methods

### Key resources table

**Table:** 

REAGENT or RESOURCE	SOURCE	IDENTIFIER
**Deposited data**		

1(a). Metadata - the FINRISK data for the present study are available with a written application to the THL Biobank as instructed on the website of the Biobank.	THL biobank	https://thl.fi/en/research-and-development/thl-biobank/for-researchers
1(b). Metadata - a separate permission is needed from FINDATA for use of the electronic health record data.	FINDATA	https://www.findata.fi/en/
2. Metagenomic data	European Genome–Phenome Archive.	https://ega-archive.org/studies/EGAS00001005020

**Software and algorithms**		

1. The source code and full list of R packages used for the analyses and figures	Github	https://github.com/finrisk2002/article-2026-gutmicrobiome-BMI-cross-sectional.

**Other**		

1. Detailed tutorial and workflow for microbiome concepts and analysis	Github	https://microbiome.github.io/OMA/docs/devel/

### Experimental model and study participant details

The FINRISK studies are population-based health examination surveys that have been carried out quinquennially in Finland since 1972. The FINRISK 2002 cohort is a stratified sample of the Finnish population aged between 25 and 74 years drawn from the national population register in 2002.^43^ The study was approved by the Coordinating Ethical Committee of the Helsinki and Uusimaa Hospital District (decision number 87/2001). All participants signed an informed consent.

#### Recruitment and study flow

Participants were recruited from six geographic regions in Finland. Before the baseline examination, the participants filled in a questionnaire on demographics, health-related behaviors, diet, as well as cardiovascular and general health. Participants then underwent a physical examination at a local study site, where height, weight, and blood pressure were recorded. Blood samples were collected after a 4-h fasting period. At the end of the baseline examination, willing participants received a stool collection kit for fecal sampling.

#### Exclusion criteria

The FINRISK 2002 study invited 13500 individuals, of whom 8798 participated in the health examination (overall participation rate 65.5%). A total of 7355 (80.2%) participants provided a stool sample for microbiome analysis. Participants were excluded from the analysis if they (i) were pregnant at the time of baseline investigation (*n* = 43); (ii) had taken antibiotics one month prior to stool sample collection (*n* = 257); (iii) had <50000 metagenome read count (*n* = 7); and (iv) had incomplete covariate data (*n* = 1202). Thus, the final sample size included in the analyses was 5846, of which 53.5% were women (Figure S1).

### Method details

#### Microbiome sampling and data processing

Stool samples were collected in 50 mL Falcon tubes and mailed overnight between Monday and Thursday to the Finnish Institute for Health and Welfare. Shipping of the samples occurred during the Finnish winter months in 2002 when outdoor temperatures are below 0°.^38^ The samples were frozen in −20° for approximately 15 years unthawed until 2017. DNA was extracted and shallow shotgun metagenomics sequencing was performed at University of California San Diego using a standard Earth Microbiome Project protocol.^44^ Samples were sequenced on Illumina Hi-Seq 4000 at 2 × 150bp read length. The computing resources of the Finnish IT Center for Science (CSC) were used for metagenome preprocessing. Sequences were then mapped to the Greengenes2 database for taxonomic profiling.^45^ Predicted pathways were annotated using MetaCyc database through the HUMAnN (v 3.0.1) pipeline.^46^^,^^47^ The taxonomic and functional profiles were imported in the R/Bioconductor microbiome framework for subsequent statistical analysis.^48^

#### Outcome variable and covariate definitions

The outcome variables of this study were obesity indicators body mass index (BMI) and waist-hip-ratio (WHR). BMI was defined as weight (kg) divided by the square of height (m). WHR was defined as waist circumference (cm) divided by hip circumference (cm). Waist circumference was measured at the midway between the lower rib margin and the iliac crest. Hip circumference was measured at the widest circumference over the greater trochanters. Measurements were rounded up to the nearest 0.5 cm.

Smoking was self-reported and categorized into two groups: 1) current smokers; and 2) non-smokers (no smoking in the past six months). Healthy food choices (HFC) were defined as a composite score from the sum of a Food Propensity Questionnaire responses to food items that ranges from 9 to 745, whereby higher scores indicate higher number of healthy food choices made per month.^49^ Prevalent cardiovascular disease and diabetes were defined using data from the nationwide Hospital Discharge Register and the Drug Purchase and Reimbursement Register (Table S1). The cardiovascular diagnoses in these registers have been validated and shown to have good coverage and accuracy.^50^^,^^51^ Alcohol consumption was self-reported and defined as grams per week. Physical activity was defined as exercise during leisure time. Participants categorized their activity level as (i) Light - “I don’t move much”, (ii) Moderate – “Walk, cycle or other form of light exercise (fishing, gardening) at least 4 h a week”, and (iii) Vigorous – “Recreational sports such as running, jogging, swimming or intense training or sports competition for at least 3 h a week”.

#### Statistical analyses

Alpha diversity indices were calculated from relative abundances at species level using the Shannon and observed richness. The association between alpha diversity and obesity indicators (BMI and WHR as continuous variable) was examined using linear regression and reported as per 1-SD change. BMI were grouped into categories according to WHO standards only for visualization. Beta diversity was calculated with supervised ordination technique dbRDA (distance-based redundancy analysis), using the Bray-Curtis dissimilarity index at the species level with relative abundance. Supervised ordination takes into account covariates when analyzing variation in the data. PERMANOVA with 999 permutations was used to assess the contribution of each covariate on microbial community dissimilarity. PERMDISP2 was utilized to assess homogeneity of the group dispersions. Beta diversity was visualized on an RDA plot with categorical BMI.

Differential abundance analyses were performed at the family, genus and species level using linear regression models. Relative abundances of microbial taxa were centered log-ratio transformed. Microbial taxa were filtered before differential abundance analyses for present in at least 5% of the population at a relative abundance of >0.1%. After filtering, 259 species, 174 genera and 52 family remained for analysis. BMI and relative abundances of microbial taxa were scaled. Thus, results are reported as 1-SD change in BMI correlates to 1-SD change in abundances. To increase robustness and decrease false positives in differential abundance analysis and predicted pathway analysis, we conducted internal validation where the cohort was split into a discovery and validation cohort. Finland is known to have differences in genetic backgrounds as well as in diet, lifestyle and general health between the east and west Finland justifying the east-west split.^52^^,^^53^ Eastern Finland (*n* = 3906) was used as a discovery cohort. Significant taxa were validated in western Finland (*n* = 1940). This approach strengthens generalizability across known sub-populations as opposed to a random 70-30 split.

Bacteroidota:Firmicutes (formerly known as Bacteroidetes) ratio was calculated by first summing all Firmicutes phyla counts. Counts were then converted to relative abundance. The ratio was calculated for each individual by dividing the relative abundance of Bacteroidota over Firmicutes.^16^ Multivariable-adjusted linear model was used to examine the association between Bacteroidota:Firmicutes ratio and BMI.

Analysis of the functions predicted based on metagenomes mapped to MetaCyc database were filtered for general pathways that were prevalent in at least 5% of the population.^46^ Metabolic pathways were not filtered by abundance to capture more pathways because shallow sequencing may result in under-represented pathways. However, so called superpathways were excluded, as they were overly general, along with species-associated pathways, and non-bacterial pathways (See sensitivity analysis). After applying the above filters, the total number of predicted pathways included in the final analysis was 343. Predicted pathway analysis was performed using linear regression models by 1) dichotomizing the pathway abundance (present versus absent), or 2) normalizing pathways using inverse rank transformation to deal with high sparsity of the pathway abundance data. As with taxa analysis, linear models were applied to eastern Finland (*n* = 3906) for discovery. Significant pathways were further validated in western Finland (*n* = 1940).

All linear models were adjusted for age, sex, diabetes, cardiovascular disease, healthy food choices, physical activity, smoking and alcohol consumption. Sex, prevalence of diabetes and cardiovascular disease, and physical activity were analyzed as categorical variables. Linear regression models’ *p* values were corrected for multiple testing using False Discovery Rate (FDR) correction and FDR <0.05 were considered statistically significant. Analyses were conducted using R version 4.3.3 and mia package 1.15.17.^48^^,^^54^ The main statistical details of each test can be found in the results section, while the full list of details can be found in Supplemental file set.

#### Sensitivity analysis

Microbial taxa were tested in association with covariates independent of BMI and added as a supplementary file to improve interpretability.

A sensitivity analysis was also conducted to identify the major bacteria species that contributed to the differences in predicted pathways. Unlike the main analysis where species-associated pathways were removed, species-associated pathways were kept for this analysis. Multivariable-adjusted linear models were used to test for differences in species-associated pathways and BMI.

#### External validation cohort

FINRISK 2007 cohort was used as an external validation cohort for the significant associations in FINRISK 2002. FINRISK 2007 cohort consisted of 275 participants. The individuals recruited in FINRISK 2007 did not participate in FINRISK 2002. Sample collection, and microbiome data processing were conducted as in FINRISK 2002.

Differences in covariates between FINRISK 2002 and 2007 were tested using Chi-square test for categorical variables and Welch’s *t* test for continuous variables. Differential abundance analyses were conducted for taxa and predicted pathways that had been internally validated in the FINRISK 2002 western cohort.

Multivariable-adjusted linear models were adjusted for age, sex, physical activity, smoking and alcohol consumption. Data for healthy food choices data were not available. Diabetes and cardiovascular disease were not included as covariates as only 0 and 3 individuals had prevalent disease, respectively. We considered *p*-values <0.05 as statistically significant as the taxa and pathways had already been internally validated.

### Additional resources

Detailed tutorial and workflow for microbiome concepts and analysis can be found here at https://microbiome.github.io/OMA/docs/devel/.

## Supplemental information

Document S1. Figures S1, S2 and Tables S1–S9

Data S1. Mean counts of bacteria family, genus and species

Data S2. Full list of significant species associated with BMI and WHR in FINRISK 2002

Data S3. List of validated species in FINRISK 2007 associated with BMI

Data S4. Full list of pathways tested in FINRISK 2002 associated with BMI

Data S5. List of validated pathways in FINRISK 2007 associated with BMI

Data S6. Full list of species associated with covariates independent of BMI
