# Supplementary material for: Population scale analysis reveals gut microbiome diversity and functional shifts linked to obesity
Source: iScience. 2026 Sep 17;29(10):117043. doi: 10.1016/j.isci.2026.117043 (PMC13602261; doi:10.1016/j.isci.2026.117043)
Supplement: Supplementary file 1 [file mmc1.pdf]

iScience, Volume *29*

Supplemental information

Population scale analysis reveals gut microbiome diversity and functional shifts linked to obesity

Li-Fang Yeo, Joonatan Palmu, Aki S. Havulinna, Dattatray Mongad, Katariina Pärnänen, Veikko Salomaa, Leo Lahti, Rob Knight, and Teemu Niiranen

Supplemental Figure SF1. Flowchart of participant inclusion

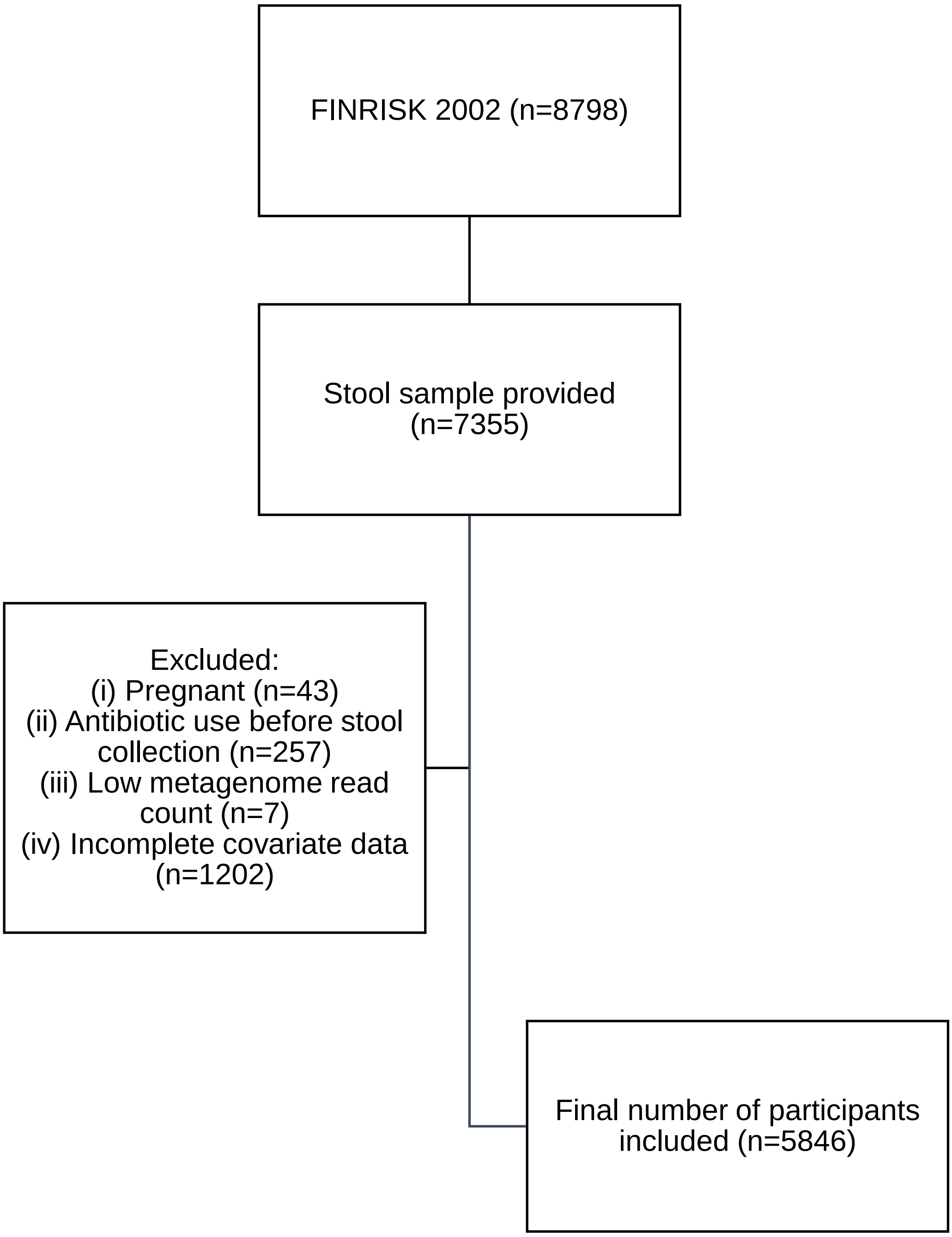

Supplemental Figure SF2. Association of taxa with BMI and waist-to-hip ratio (WHR) in FINRISK 2002.

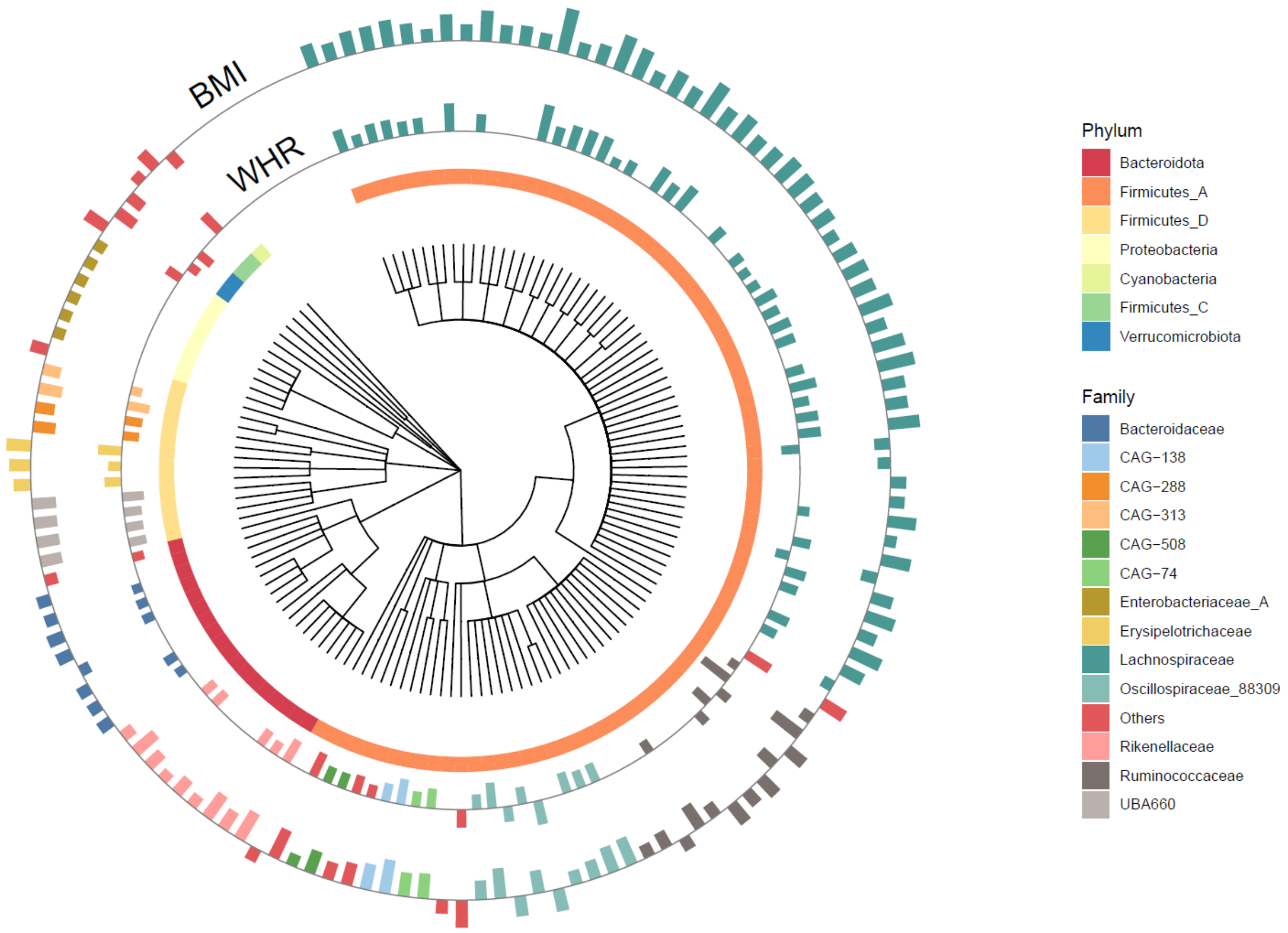

The innermost circle depicts the phylum of the significant taxa. The bars on the two outer circles represent the linear model beta estimates per 1-SD changes in taxa abundance for BMI and WHR. Upon internal validation within FINRISK 2002 cohort, 132 and 105 species, as depicted in this figure, remained significant for BMI and WHR respectively (FDR < 0.05).

This figure shows that many of the significant taxa overlap between BMI and WHR, hence justifying the subsequent focus of the study on only BMI. A large proportion of positively associated taxa belong to the Firmicutes A phylum, Lachnospiraceae family.

Bacteria family that appears only once were labelled as “Others”. Linear regression model was adjusted for age, sex, diabetes, cardiovascular disease, healthy food choices, physical activity, smoking and alcohol consumption.

Supplemental Table ST1. Definitions of register-based comorbidities and outcomes.

Comorbidity/Outcome	Hospital discharge and death registers	Drug reimbursement and purchase registers
Diabetes	ICD-10: E10-E14<br>ICD-9: 250<br>ICD-8: 250	ATC: A10, A10A, A10B<br>DR: 103
Cardiovascular disease (*As a primary or secondary diagnosis code)	Hospital discharge register:<br>CD-10: I200, I21*, I22*, I61, I63* (not I63.6), I64*<br>ICD-9: 410*, 4110, 431*, 4330A*, 4331A*, 4339A*, 4340A*, 4341A*, 4349A*, 436*<br>ICD-8: 410* 4110, 431* (not 43101 or 43191) 433*, 434*, 436*<br>Procedure code for PCI or CABG<br><br>Causes of death register:<br>ICD-10: I20-I25, I21-I22* I46, R96, R98, I61*, I63* (not I63.6), I64*<br>ICD-9: 410-414, 410*, 798 (not 7980A), 431*, 4330A*, 4331A*, 4339A*, 4340A*, 4341A*, 4349A*, 436*<br>ICD-8: 410-414, 798 (not 7980A), 431* (not 43101 or 43191) 433*, 434*, 436*	

ICD, International Classification of Diseases; ATC, Anatomical therapeutic chemical code; CABG, coronary artery bypass grafting; DR, Drug reimbursement code; PCI, percutaneous coronary intervention.

Supplemental Table ST2 Linear model statistics of alpha diversity (Shannon) and waist-hip-ratio (WHR).

Predictor	Estimate	SE	Statistic	P	conf.low	conf.high
(Intercept)	-0.49	0.02	-22.21	<0.001	-0.53	-0.44
Shannon	-0.04	0.01	-4.85	<0.001	-0.06	-0.03
Age	0.22	0.01	23.24	<0.001	0.20	0.23
Sex	1.33	0.02	69.99	<0.001	1.29	1.37
Diabetes	0.35	0.05	7.83	<0.001	0.27	0.44
Cardiovascular disease	0.09	0.05	1.77	0.077	-0.01	0.19
Smoking	0.03	0.02	1.39	0.165	-0.01	0.07
Alcohol consumption	0.06	0.01	6.35	<0.001	0.04	0.08
Healthy food choices score	-0.01	0.01	-0.73	0.468	-0.03	0.01
Leisure-time exercise, moderate	-0.13	0.02	-5.70	<0.001	-0.17	-0.08
Leisure-time exercise, vigorous	-0.34	0.03	-12.70	<0.001	-0.40	-0.29

SE, standard error

Continuous variables are reported as per 1-SD increase.

Supplemental Table ST3 Linear model statistics of observed species richness and waist-hip-ratio (WHR).

Predictor	Estimate	SE	Statistic	P	conf.low	conf.high
(Intercept)	-0.49	0.02	-22.16	<0.001	-0.53	-0.44
Observed richness	0.01	0.01	1.09	0.275	-0.01	0.03
Age	0.21	0.01	22.99	<0.001	0.20	0.23
Sex	1.33	0.02	70.06	<0.001	1.30	1.37
Diabetes	0.36	0.05	7.85	<0.001	0.27	0.44
Cardiovascular disease	0.09	0.05	1.70	0.089	-0.01	0.18
Smoking	0.03	0.02	1.63	0.104	-0.01	0.08
Alcohol consumption	0.06	0.01	6.46	<0.001	0.04	0.08
Healthy food choices score	-0.01	0.01	-0.96	0.337	-0.03	0.01
Leisure-time exercise, moderate	-0.13	0.02	-5.84	<0.001	-0.18	-0.09
Leisure-time exercise, vigorous	-0.35	0.03	-12.86	<0.001	-0.40	-0.30

SE, standard error

Continuous variables are reported as per 1-SD increase.

Supplemental Table ST4. Microbial compositional differences (beta diversity) for BMI using PERMANOVA

	Df	SumOfSqs	F	Pr(>F)	Total variance	Explained variance (R^2)
Model	10	21.32	11.45	0.001	1108.11	0.019
Age	1	3.70	19.85	0.001	1108.11	0.003
Sex	1	4.44	23.82	0.001	1108.11	0.004
Diabetes	1	0.65	3.46	0.001	1108.11	<0.001
Cardiovascular disease	1	0.26	1.38	0.106	1108.11	<0.001
Smoking	1	1.75	9.40	0.001	1108.11	0.002
Alcohol consumption	1	1.48	7.93	0.001	1108.11	0.001
Healthy food choices	1	1.05	5.66	0.001	1108.11	0.001
Physical activity	2	0.89	2.40	0.001	1108.11	<0.001
BMI	1	3.67	19.70	0.001	1108.11	0.003
Residual	5835	1086.79	NA	NA	1108.11	0.98

Df, degree of freedom, SumofSqs, sum of squares, BMI, body-mass index

Supplemental Table ST5. Microbial compositional differences (beta diversity) for WHR using PERMANOVA

	Df	SumOfSqs	F	Pr(>F)	Total variance	Explained variance (R^2)
Model	10	20.02	10.82	0.001	1099.923	0.018
Age	1	3.92	21.20	0.001	1099.923	0.004
Sex	1	2.51	13.56	0.001	1099.923	0.002
Diabetes	1	0.62	3.33	0.001	1099.923	0.001
Cardiovascular disease	1	0.37	2.00	0.006	1099.923	0.000
Smoking	1	1.59	8.57	0.001	1099.923	0.001
Alcohol consumption	1	1.38	7.47	0.001	1099.923	0.001
Healthy food choices	1	1.04	5.60	0.001	1099.923	0.001
Physical activity	2	0.82	2.20	0.001	1099.923	0.001
WHR	1	2.40	12.95	0.001	1099.923	0.002
Residual	5835	1079.91	NA	NA	1099.923	0.982

Df, degree of freedom, SumofSqs, sum of squares, WHR, waist-hip ratio

Supplemental Table ST6. Linear model statistics for Bacteroidota:Firmicutes ratio associated with BMI.

	Estimate	SE	t-value	P
(Intercept)	-0.682	0.063	-10.777	<0.001
Bacteroidota:Firmicutes ratio	0.002	0.014	0.124	0.901
Age	0.018	0.001	17.562	<0.001
Sex	0.132	0.027	4.836	<0.001
Diabetes	0.449	0.065	6.933	<0.001
Cardiovascular disease	-0.052	0.072	-0.722	0.47
Smoking	-0.142	0.031	-4.604	<0.001
Alcohol consumption	0.0002	0.0001	2.061	0.039
Healthy food choices	-0.0001	0.0002	-1.115	0.265
Physical activity – moderate	-0.231	0.032	-7.095	<0.001
Physical activity – vigorous	-0.473	0.039	-12.205	<0.001

BMI, body mass index.

Bacteroidota is an updated name for Bacteroidetes. Estimates are reported as per 1-SD change.

Supplemental Table ST7. Linear model statistics for Bacteroidota:Firmicutes ratio associated with WHR.

	Estimate	SE	t value	P
(Intercept)	-1.331	0.044	-30.079	<0.001
Bacteroidota:Firmicutes ratio	0.005	0.009	0.553	0.58
Age	0.017	0.001	23.045	<0.001
Sex	1.334	0.019	70.017	<0.001
Diabetes	0.355	0.045	7.833	<0.001
Cardiovascular disease	0.086	0.05	1.721	0.085
Smoking	0.034	0.021	1.577	0.115
Alcohol consumption	<0.001	<0.001	6.421	<0.001
Healthy food choices	<0.001	<0.001	-0.935	0.35
Physical activity - moderate	-0.132	0.023	-5.824	<0.001
Physical activity - vigorous	-0.348	0.027	-12.85	<0.001

WHR, waist-hip ratio.

Bacteroidota is an updated name for Bacteroidetes. Estimates are reported as per 1-SD change

Supplemental table ST8. Characteristics table comparing the original cohort to the external validation cohort

	Original cohort	Validation cohort	P
Name	FINRISK 2002	FINRISK 2007	
N	5846	275	
Age, years (SD)	48.6 (12.8)	36.0 (5.6)	<0.001
Women, n (%)	2705 (46.3)	120 (43.6)	0.43
BMI, kg/m^2 (SD)	26.8 (4.6)	25.9 (3.6)	<0.001
Waist circumference, cm (SD)	89.3 (13.4)	88.0 (12.1)	0.11
Waist-hip ratio (SD)	0.90 (0.09)	0.89 (0.09)	0.16
Diabetes, n (%)	229 (3.9)	0 (0.0)	0.001
Cardiovascular disease, n (%)	189 (3.2)	3 (1.1)	0.07
Smoking, n (%)	1400 (23.9)	54 (19.6)	0.12
Alcohol consumption, grams/week (SD)	83.1 (123.7)	65.2 (80.2)	0.02
Healthy food choices score (SD)	196.66 (88.22)	NA	NA
Leisure-time exercise			<0.001
Light, n (%)	1198 (20.5)	55 (20.0)	
Moderate, n (%)	3274 (56.0)	107 (38.9)	
Vigorous, n (%)	1374 (23.5)	113 (41.1)	
Shannon diversity (SD)	4.12 (0.43)	3.98 (0.44)	<0.001
Observed richness (SD)	1062.8 (215.3)	1024.2 (158.9)	0.003
Total reads (SD)	1055378 (984488)	NA	NA

SD, standard deviation; BMI, body mass index

*Higher healthy food choices score indicates more healthy food choices made per month.

Continuous variables are tested using t-test, categorical variables are tested using chi-square test. Healthy food choices score and total reads were not available for FINRISK 2007 cohort.

Supplemental table ST9. Top ten species that contributed to 89% of the significant pathways.

Species	No. of associated pathways	Depleted / Enriched	Significant in taxa-level analysis?
Eubacterium rectale	46 (all enriched)	Enriched in carbohydrate biosynthesis pathway	No
Bacteroides vulgatus	53 (all enriched)	Enriched in biosynthesis-related pathways	Yes, enriched in higher BMI
Ruminococcus torques	43 (all enriched)	Enriched in amino acid biosynthesis-related pathways	Yes, enriched in higher BMI
Butyrivibrio crossotus	37 (all depleted)	Depleted in biosynthesis-related pathways	Yes, depleted in higher BMI
Alistipes finegoldii	22 (all depleted)	Depleted in starch degradation, nucleoside salvage and fermentation pathways	Yes, depleted in higher BMI
Fusicatenibacter saccharivorans	30 (all enriched)	Enriched in amino acid biosynthesis-related pathways	Yes, enriched in higher BMI
Lachnospira pectinoschiza	7 (all enriched)	Enriched in nucleotide synthesis pathways	No
Roseburia faecis	20 (all enriched)	Enriched in amino acid biosynthesis-related pathways	Yes, enriched in higher BMI
Roseburia inulinivorans	13 (all enriched)	Enriched in carbohydrate-related pathways	Yes, enriched in higher BMI
Anaerostipes hadrus	9 (all enriched)	Enriched in biosynthesis pathways	No

Species are arranged approximately from smallest to largest FDR value.
